# Comparing Phylogeographies to Reveal Incompatible Geographical Histories within Genomes

**DOI:** 10.1093/molbev/msae126

**Published:** 2024-06-26

**Authors:** Benjamin Singer, Antonello Di Nardo, Jotun Hein, Luca Ferretti

**Affiliations:** Department of Medicine, Stanford University, Stanford, CA, USA; The Pirbright Institute, Pirbright, Woking, Surrey, UK; Department of Statistics, University of Oxford, Oxford, UK; Pandemic Sciences Institute, Nuffield Department of Medicine, University of Oxford, Oxford, UK; Big Data Institute, Li Ka Shing Centre for Health Information and Discovery, Nuffield Department of Medicine, University of Oxford, Oxford, UK

**Keywords:** phylogeography, recombination, viral evolution

## Abstract

Modern phylogeography aims at reconstructing the geographic movement of organisms based on their genomic sequences and spatial information. Phylogeographic approaches are often applied to pathogen sequences and therefore tend to neglect the possibility of recombination, which decouples the evolutionary and geographic histories of different parts of the genome. Genomic regions of recombining or reassorting pathogens often originate and evolve at different times and locations, which characterize their unique spatial histories. Measuring the extent of these differences requires new methods to compare geographic information on phylogenetic trees reconstructed from different parts of the genome. Here we develop for the first time a set of measures of phylogeographic incompatibility, aimed at detecting differences between geographical histories in terms of distances between phylogeographies. We study the effect of varying demography and recombination on phylogeographic incompatibilities using coalescent simulations. We further apply these measures to the evolutionary history of human and livestock pathogens, either reassorting or recombining, such as the Victoria and Yamagata lineages of influenza B and the O/Ind-2001 foot-and-mouth disease virus strain. Our results reveal diverse geographical paths of migration that characterize the origins and evolutionary histories of different viral genes and genomic segments. These incompatibility measures can be applied to any phylogeography, and more generally to any phylogeny where each tip has been assigned either a continuous or discrete “trait” independent of the sequence. We illustrate this flexibility with an analysis of the interplay between the phylogeography and phylolinguistics of Uralic-speaking human populations, hinting at patrilinear language transmission.

## Introduction

The study of the evolutionary biology and phylodynamics of pathogens has made significant contributions to disease control over the last few decades (Baele et al. 2018; Grubaugh et al. 2019; Markov et al. 2023). Pathogens are dispersed with their human and animal hosts, both of which are seeing increasing mobility as an effect of global tourism, the access of emerging economies to international markets, and climate change (Gushulak and MacPherson 2000; Saker et al. 2004; Carlson et al. 2022). These conditions offer increasing opportunities for some pathogens to spread rapidly at the global level. Seasonal and pandemic influenza (Lemey et al. 2014) and more recently the SARS-CoV-2 pandemic (Kraemer et al. 2020; McCrone et al. 2022) are well known examples. Increased mobility combines with high evolution rates in some pathogens, coupling the processes of evolution and spatial migration. To understand the emergence, adaptation and spread of these pathogens, therefore, we must use the methods provided by the field of modern phylogeography, that jointly infer the evolutionary history (i.e. phylogeny) and the spatial history (i.e. geographic migration of an organism Dudas et al. 2017).

Phylogeographic methods can make use of the mathematical representation of a phylogeny as a tree, allowing the spatial evolution from the root to the tips to be seen as a migration process. This migration process is assumed to proceed independently on each branch of the tree, with a single origin at the root. The resulting structure greatly simplifies mathematical and computational treatments of systems composed of coupled evolution and migration processes. However, the phylogenetic tree of a set of DNA or protein sequences contains information not only on the relatedness of sequences, but also on their evolutionary history. There exist many different methods for reconstructing such a tree. If genome isolates have been collected from different places, their history could be affected by the geographic structure of the populations, including the rates of migration across different locations (Lemey et al. 2014). The first step to understanding these effects is the reconstruction of a phylogenetic tree with geographical information attached to the internal nodes, obtained through reconstruction of the spatial movements of lineages in the tree. Such an object corresponds to the phylogeographic history of the given set of sequences. For simplicity, in this paper we will refer to it as a *phylogeography*.

A range of techniques have been developed for the reconstruction of phylogeographies (Faria et al. 2011). These can be classified according to how the migration process of ancestral lineages is modeled on the tree: either in the form of instantaneous migration of discrete traits (such as finite spatial locations), i.e. *discrete phylogeography* based on the coalescent (Lemey et al. 2009), structured coalescent (Vaughan et al. 2014; De Maio et al. 2015; Müller et al. 2017, 2018) or birth–death processes (Kühnert et al. 2016; Scire et al. 2022); or as relaxed random walk movement in two-dimensional geographic space, i.e. *continuous phylogeography* (Lemey et al. 2010; Bouckaert 2016; Dellicour et al. 2016; Gill et al. 2017). Many of these approaches focus on a single tree, obtained from concatenation-based phylogeny or selected sections of a genome, and therefore do not take into account the decoupling of the evolutionary history of different genomic regions due to recombination and/or related processes. Phylogeography and analogs have received a lot of interest lately, clearly motivated by the serious threats to public health from infectious diseases such as seasonal flu and SARS-CoV-2. Approaches focused on extending structured coalescent to account for ancestral recombination have been recently published: Guo et al. (2022) used a general probabilistic model of structured coalescent that explicitly includes recombination in the genealogical process, whilst Stolz et al. (2022) focused on reassortment, a process occurring in viruses with segmented genomes such as influenza that leads to exchange of discrete genomic segment.

For many species of eukaryotes, as well as for some viruses and bacteria, phylogenies are not tree-like. In the presence of recombination, reassortment, or horizontal gene transfer, different regions of the genome might have potentially evolved from unique ancestors. This decouples the evolutionary histories of different genes or regions of the genome, resulting in trees with cross-branch reticulations if the number of recombination events is small, or in phylogenetic networks if recombination is widespread (Edwards et al. 2016). Recombination leads to organisms that inherit genomic fragments from multiple parent lineages, causing phylogenetic inference methods applied to different parts of the genome to give rise to incompatible trees. By applying a measure of *tree distance* to these trees, it is possible to make certain claims about the amount of recombination in the ancestral population (Chung et al. 2013), for instance by setting a lower bound on the number of recombination events between loci. Ignoring the role of inference errors, any nonzero tree distance reveals an incompatibility. If the genealogies of different parts of the same genome are incompatible, these incompatibilities can be evidence of recombination, horizontal gene transfer, reassortment, or gene conversion. A variety of incompatibility measures and tree distances exist for phylogenies, most notably the well-known Robinson–Foulds metric and the family known as tree edit (or Levenshtein) distances (Day 1984), including the Subtree-Prune-and-Regraft (SPR) distance (Semple and Steel 2003).

Recombination can lead to a decoupling of geographic histories between loci (Hare 2001), just as it can lead to a decoupling of phylogenetic histories. The resulting ensemble of spatial trajectories may reveal functional and evolutionary features of different genes. However, phylogeographic inference is highly nontrivial in the presence of reticulations. Reticulations form phylogenetic loops, meaning that two lineages can split at some time in the past, move in geographic space, then converge to the same location and combine into a single lineage. They can share pieces of genetic material with different geographical origins, or copies of material which have reached their current geographical distribution and evolutionary trajectory in different ways. This means that the spatial evolution of a given pair of lineages can no longer be assumed independent, and some of the advantages of working with migration processes on a tree are lost.

In this article, we define two mathematical measures of the degree of difference between reconstructed phylogeographies arising from multiple loci. Only one of these is a true *distance* in the sense of providing a metric on a vector space, so we will use the more general term “incompatibility measure.” A nonzero value of an incompatibility measure between two phylogeographies implies that they are incompatible, and could be evidence of recombination. Comparing incompatibilities between the phylogeographies of different loci can reveal which regions have highly divergent histories, and conversely which regions tend either not to recombine, or to remain associated during spatial migration. We apply these measures of phylogeographic incompatibility to reconstructed histories of a reassorting human pathogen, the influenza B virus, as well as a recombining animal pathogen, the foot-and-mouth disease virus (FMDV). In addition, we further illustrate potential interdisciplinary applications to other types of data by analysing the phylogenetic, phylogeographic, and phylolinguistic landscape of Uralic-speaking populations.

## New Approaches

Any measure of phylogeographic incompatibility must be related to phylogenetic distances, due to the mathematical relation between phylogeographies and phylogenies (explained below). In this section, we outline our approach to calculating phylogeographic incompatibilities, starting with a comparison to the phylogenetic case and detailing two possible ways to incorporate geographic information. We then provide an approach that accounts for uncertainties in phylogeographic reconstruction.

### Phylogenetic Incompatibilities

Many different measures of tree distance/incompatibility have been developed in the past. In most comparisons, we will use only two measures that are conceptually related to the phylogeographic incompatibilities introduced in the next sections.

The first incompatibility measure is the Kendall–Colijn metric (“KC”) (Kendall and Colijn 2016). This Euclidean vector-based tree distance metric compares the height of the most recent common ancestors (MRCAs) of each pair of leaves (i.e. terminal nodes, tips, or taxa) on rooted trees.

The second is the distance based on the size of the maximum agreement sub-tree (“MAST”) between the two trees (this concept is explained further in the subsection titled Maximum Agreement Sub-Phylogeography below) (Steel and Warnow 1993). The MAST estimate is very large for similar trees, while it involves fewer and fewer leaves as the dissimilarity between the trees increases. Note that the normalized MAST distance is usually defined as the fraction of leaves that do *not* belong to the MAST.

We also consider four other distance measures defined on phylogenies (without a geographical element):

- Robinson–Foulds metric (“RF”) (Robinson and Foulds 1981)
- Robinson–Foulds metric weighted by branch lengths (“WRF”) (Robinson and Foulds 1979)
- Subtree-Prune-and-Regraft edit distance (“SPR”) (Allen and Steel 2001; De Oliveira Martins et al. 2014)
- Kuhner–Felsenstein branch score distance (“KF”) (Kuhner and Felsenstein 1994)

For each of these incompatibility measures, a version exists that defines a proper metric in the space of either rooted or unrooted binary trees with branches of finite length (Goddard et al. 1994). However, all these distances capture different ways in which phylogenies can differ (Smith 2022). Given the high-dimensional and heterogeneous nature of phylogenetic spaces, it is not surprising that different measures give different results, as illustrated in Supplementary material online. As an example, trees differing in topology near the root and especially in tree balance tend to have a very large KC distance (Smith 2022). Single recombination events near the root can have a dramatic impact on the MAST, while recombination events involving lower branches or tips have a small effect on the MAST, even if they causes changes in the root.

Throughout this paper, we used the version of these measures adapted to unrooted binary trees, with the exception of KC. These measures are currently implemented in the R packages *APE* (Paradis and Schliep 2018), *phangorn* (Schliep 2010), and *treespace* (Jombart et al. 2017).

### Phylogeographic Space

The first step in building natural methods of comparison for phylogeographies is to define what a phylogeography is and to situate it in a well-defined mathematical space. The incompatibility measures should facilitate the definition of distance measures over this space. Such a space needs to be able to represent both phylogenetic and geographical information.

We define a phylogeography as a tree with geographical information assigned to each node of the tree. For practical purposes, inferred phylogenies are represented by rooted phylogenetic trees with branch lengths and labeled tips. Geographical space can be represented either as a network of discrete locations or as a continuous space. Our approach covers both cases. Two questions then arise: how to combine the genetic distances in the phylogenetic trees with the geographic distances, and how to define a joint measure on the resulting combination. These two questions are not independent, as different spaces comprising different kinds of mathematical objects require different approaches for comparing phylogeographies.

**Fig. 1.**
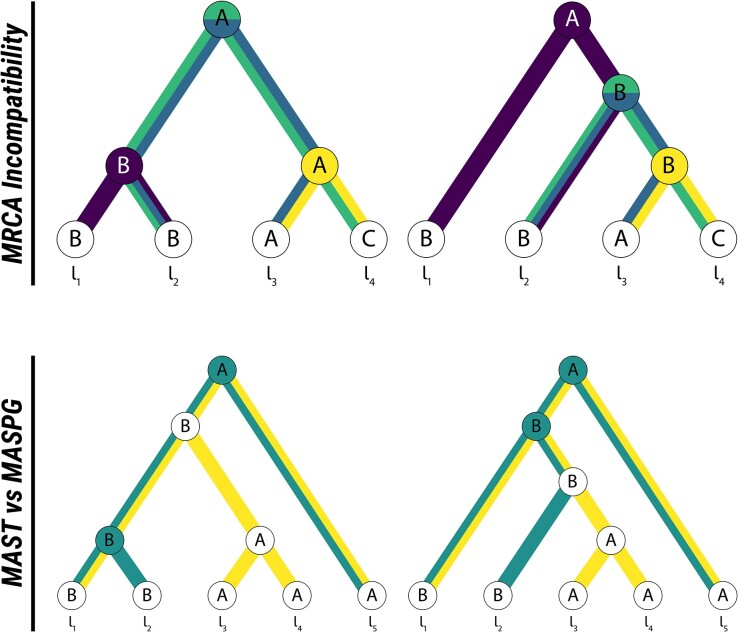
An illustration of the two measures of phylogeographic incompatibility presented in this paper: (Upper Panel) two phylogeography diagrams illustrating the MRCA-based incompatibility measure. Paths to MRCA of pairs of leaves with conflicting locations are highlighted in colors corresponding to leaf pairs (purple, $\left\{l_{1},l_{2}\right\}$; blue, $\left\{l_{2},l_{3}\right\}$; green, $\left\{l_{2},l_{4}\right\}$; yellow, $\left\{l_{3},l_{4}\right\}$). The nodes causing the incompatibility are likewise highlighted. There are four conflicting MRCAs here, so the unweighted incompatibility is 4. (Lower Panel) Two phylogeography diagrams illustrating the difference between the MAST (in yellow) and the MASPG (in green). The MASPG includes internal node locations, and the internal nodes that contribute to the MASPG are highlighted in green. Due to the requirement of matching internal node locations, the MASPG has size 3, while the MAST, which does not have this constraint, has size 4.

We present a simple definition of phylogeographic space. The most general space is denoted by ${PG}_{n}$, with integer *n*, and its elements are rooted binary trees with *n* labeled leaves and with locations assigned to all nodes (both leaves and internal nodes). The illustrations in Fig. 1 are of phylogeographies of this kind. This space corresponds to the Cartesian product of the space of bifurcating rooted trees with *n* leaves $T_{n}$ and the Cartesian power of the geographic space G to the number of nodes, i.e. ${PG}_{n}=T_{n}\times G^{2n-1}$. In practice, we will work on subspaces of ${PG}_{n}$ with a given vector of tip locations g, since the locations of the leaves are usually known and given as input into phylogeographic analyses. For a given set of locations g, the corresponding phylogeographic subspace ${PG}_{g}$ is isomorphic to $T_{n}\times G^{n}$. In the following, when discussing ${PG}_{g}$ for a specific (although implicit) choice of g, we will refer to it simply as PG.

### Pairwise MRCA Incompatibilities

We assume that the geographic space G has a metric $d_{G}(g,g^{\prime })$ already defined on it for phylogeographic analyses—for example, it can be the geographical distance, the distance between centroids of discrete areas (e.g. countries and other political units), or the shortest path distance in the case of a mobility network. We can use this distance to define a purely geographic measure of incompatibilities on PG. However, this requires a pairwise comparison of spatial locations. Since locations are assigned to nodes on different trees, we must solve the matching of nodes across trees with different shapes. A straightforward approach is to map the MRCAs for pairs of leaves, i.e. compare nodes which are the MRCA of the same pair of leaves on different trees.

The first step to performing such a comparison for a pair of trees with *n* leaves is to project the phylogeography *PG* to a (n2)-ary vector $g_{MRCA}(PG)$, where the element $g_{MRCA(l_{1},l_{2})}$ is the location of the MRCA of the tips $l_{1}$ and $l_{2}$. Such vectors span a subspace of the (n2)-ary Cartesian power of G. The $L_{1}$ product metric is a natural metric for this space, and therefore it can be used to compute geographic incompatibilities between phylogeographies.

The incompatibility measure between two phylogeographies $PG_{1}$ and $PG_{2}$ is then defined as


(1) $$I_{MRCA}(PG_{1},PG_{2})=\frac{\sum_{\left\{l_{1},l_{2}\right\}}d_{G}(g_{MRCA(l_{1},l_{2})}(PG_{1}),g_{MRCA(l_{1},l_{2})}(PG_{2}))}{\left(\begin{array}{c} n\\ 2 \end{array}\right)},$$

where $\left\{l_{1},l_{2}\right\}$ range over the set of pairs of leaves, $g_{MRCA(l_{1},l_{2})}(PG_{k})$ is the location assigned to the MRCA of leaves $l_{1}$ and $l_{2}$ in the phylogeography $PG_{k}$, and $d_{G}$ is the metric on the geographic network. The normalization factor (n2) ensures that the minimum and maximum possible values of the measure are 0 and $\max_{g,g^{\prime }}d_{G}(g,g^{\prime })$, respectively.

For a simple example of how to calculate $I_{MRCA}$, consider the phylogeographies in Fig. 1. Take *A*, *B*, and *C* to refer to the arbitrary locations Argentina, Bolivia, and Chile, and place these countries on a network where there is a pairwise distance of 1 between each country, since they all share borders. The MRCA vectors are therefore $g_{MRCA}(PG_{1})=(B,A,A,A,A,A)$ for the phylogeography on the left, and $g_{MRCA}(PG_{2})=(A,A,A,B,B,B)$ for the phylogeography on the right. So the total incompatibility is $I_{MRCA}(PG_{1},PG_{2})=\frac{4d_{G}(A,B)}{6}=2/3$. Here, the incompatibility comes about due to four comparisons between Argentina and Bolivia, and the only MRCA paths that do not contribute to the incompatibility are between $l_{1}$ and $l_{3}$, and between $l_{1}$ and $l_{4}$, which meet at the root in both phylogeographies.

$I_{MRCA}$ is especially sensitive to the locations assigned to nodes with many descendants, the strongest example of this being the root of the tree, which can contribute to up to half of the comparisons made in calculating the incompatibility. This sensitivity depends in part on the tree shape, with the root contributing more in symmetric trees than in asymmetric ones. This is because leaf pairs are more likely to find their MRCA at the root when the tree is more symmetric, and more likely to find their MRCA on the trunk of the tree when the tree is less symmetric.

Note that $I_{MRCA}$ is not a distance on PG, since trees with a different topology may still have no incompatibilities. However, it is easy to construct a distance on PG using $I_{MRCA}$; in fact, any positive linear combination of $I_{MRCA}$ and a tree distance on $T_{n}$ is a proper metric on PG (see the Supplementary material online for proofs that incompatibility measures are distances on PG and further discussions on their properties).

### Maximum Agreement Sub-Phylogeography

To incorporate both phylogenetic and geographic differences into a metric in a unified way, an alternative approach is to extend an existing metric on $T_{n}$ into ${PG}_{n}$. One of the measures of phylogenetic incompatibility is based on the notion of a maximum agreement subtree (MAST), which we will here extend to phylogeographies.

There exists a comprehensive body of literature on finding MASTs between pairs of trees (Hein et al. 1996; Semple and Steel 2003; Steel 2016). An agreement subtree between the trees $T_{1}$ and $T_{2}$ with labeled leaves is a tree *S*, with labeled leaves, such that the subtrees induced on $T_{1}$ and $T_{2}$ by the set of leaves corresponding to those in *S* (i.e. the subtrees containing only those leaves and internal nodes) are both identical to *S*. The MAST is the largest such subtree. Figure 1 provides an example of a MAST.

We can use the MAST to define a metric over $T_{n}$ (Steel and Warnow 1993). To find the distance between $T_{1}$ and $T_{2}$, we start to find $MAST(T_{1},T_{2})$, and then write $d_{MAST}(T_{1},T_{2})=n-|MAST(T_{1},T_{2})|$. Here, |T| is the number of leaves on *T*, and $n=|T_{1}|=|T_{2}|$. Thus, for the trees in the example in Fig. 1, $d_{MAST}(T_{1},T_{2})=1$, since the trees are of size five and the MAST is of size four.

Now we extend this distance to a metric on PG. First, we define a maximum agreement sub-phylogeography (MASPG). Take two phylogeographies $PG_{1}$ and $PG_{2}$, with locations assigned to each node. Let us define an agreement sub-phylogeography as a sub-phylogeography without unary nodes such that (i) the corresponding subtree is an agreement subtree and (ii) for each node in the subtree, the location assigned to the node matches the locations on the corresponding subtrees in both $PG_{1}$ and $PG_{2}$. That is, the induced subtrees must be identical not only in topology but also in the geographical assignment of the nodes. The MASPG is defined as the largest such sub-phylogeography.

We can then define the phylogeographic incompatibility between two phylogeographies $PG_{1}$ and $PG_{2}$, both with leaf number $n=|PG_{1}|=|PG_{2}|$, as


(2) $$I_{MASPG}(PG_{1},PG_{2})=1-|MASPG(PG_{1},PG_{2})|/n,$$

where |T| is the number of leaves on the tree *T*. With this normalization, the minimum and maximum possible values of $I_{MASPG}$ are 0 and 1, respectively. For the phylogeographies in Fig. 1 the MASPG is of size 3 and the phylogeographies are of size 5, so the incompatibility is 1−3/5=0.4.

### Incompatibility Between Distributions of Phylogeographies

In practical applications, phylogeographies are not known with absolute precision, but they are inferred from noisy and biased data (e.g. biased sampling of subpopulations). In fact, Bayesian approaches to phylogeographic inference actually return a posterior distribution of phylogeographies. For this reason, we consider the more general case of comparisons between two *distributions* of phylogeographies. In such a case, the objects of comparison are two random variables ${\mathrm{PG}}_{1}$ and ${\mathrm{PG}}_{2}$ derived from different loci along a genome. These variables are likely to be highly correlated even if their posterior (marginal) distributions are inferred independently, due to linkage between loci and the fixed assignment of tip locations from sample metadata. This causes strong biases if the average value of $I_{MRCA}$ on the posterior distributions is computed assuming the two distributions as independent.

The most conservative choice is to compute the incompatibility as a Wasserstein metric (or earth mover’s distance) between the distributions, using one of the incompatibility measures defined above as the cost function. This choice reduces the likelihood of spurious incompatibilities due to correlations between the inferred phylogeographies at different loci. As an example, if the two loci have identical posterior distributions, this would result in a nonzero distance defining the distributions as independent, while the Wasserstein metric implicitly assumes maximum dependence and therefore results in zero distance, i.e. full phylogeographic compatibility.

Hence, we can use $I_{MRCA}$ or $I_{MASPG}$ as a cost function for differences between phylogeographies, yielding a definition of incompatibility between ${\mathrm{PG}}_{1}$ and ${\mathrm{PG}}_{2}$ as the first Wasserstein metric on the corresponding distributions. In practice, we estimate this by taking *N* samples from each posterior, pairing these samples in all possible N! ways (e.g. $s_{1}$ with $s_{1}^{\prime }$, $s_{2}$ with $s_{2}^{\prime }$ etc.), computing the mean of the incompatibilities $\sum_{i=1}^{N}I(s_{i},s_{i}^{\prime })/N$ between all pairs of samples from the two distributions, then minimizing this quantity over all N! choices of pairings. This is an assignment problem that can be computed by classical linear programming algorithms. In Table 1, we compare the Wasserstein distance computed on a random sample of 10 trees obtained from the full posterior with the distance computed on a single summary phylogeography for the examples of the pathogen phylogenies presented in the next sections.

**Table 1:** Comparison between the incompatibilities computed on the MCC tree and on a random sample of 10 trees obtained from the posterior distributions

Tree Data	Gene Comparison	Metric	MCC Distance	Wasserstein distance (SD)
FMDV	VP1 vs 3D	KC	163	115 (13)
		MAST	0.436	0.450 (0.009)
		MRCA	0.359	0.299 (0.076)
		MASPG	0.656	0.642 (0.029)
Influenza B - Victoria	HA vs NA	KC	186	182 (4)
		MAST	0.601	0.624 (0.008)
		MRCA	0.358	0.462 (0.056)
		MASPG	0.796	0.824 (0.017)
Influenza B - Yamagata	HA vs NA	KC	142	151 (5)
		MAST	0.528	0.548 (0.010)
		MRCA	0.406	0.523 (0.084)
		MASPG	0.742	0.788 (0.023)

It is also possible to use a similar approach to evaluate the impact of the uncertainties in phylogenetic reconstruction, as discussed in Supplementary material online.

## Results

### Effect of Evolutionary Parameters on Phylogeographic Incompatibilities

To understand the impact of recombination and migration rates on phylogeographic incompatibilities, we generated random phylogeographies from structured coalescent models with three populations/locations. We simulated both coalescent trees and node locations of 20 sequences from each location, all sampled at the present time. Simulations were performed by varying population-scaled recombination rate *ρ* and migration rate *μ*, as well as migration patterns.

**Fig. 2.**
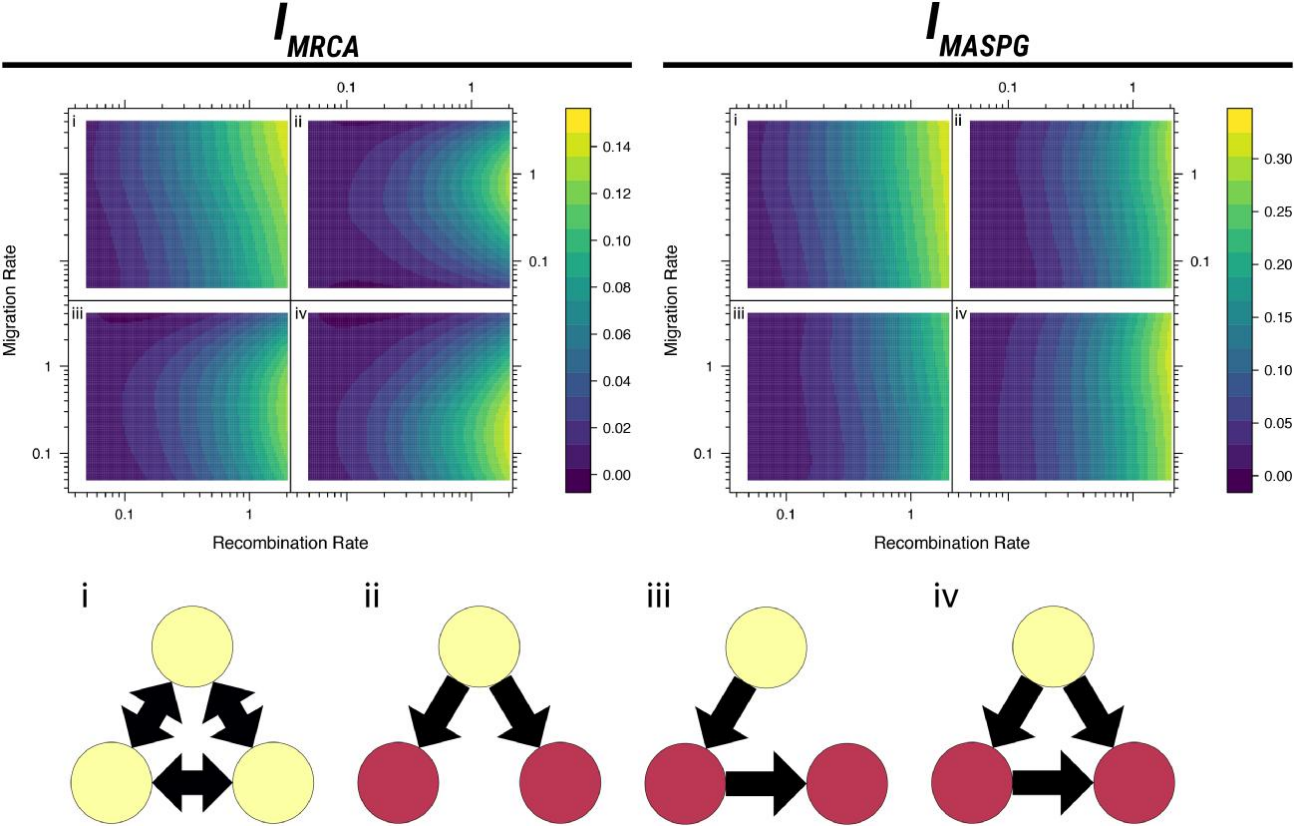
Upper panel: Effect of evolutionary parameters on phylogeographic incompatibilities, using 100 simulations at each calculated pair of parameter values. The plots show smoothed surfaces of each phylogeographic incompatibility measure, labeled with the corresponding network structure. Lower panel: Models of the network structures. The network structures are illustrated with locations in which the population can originate colored in yellow.

Incompatibilities are generated by different mechanisms for the patterns of migration represented in the diagrams shown in the lower panel of Fig. 2. In model (iii), there is only one possible chain of geographical transmissions for each lineage, but incompatibilities are generated by the different pace of lineages along this chain. These differences originate from neutral (stochastic) or selective differences in the patterns of coalescence or migration of the lineages. In model (iv), multiple patterns of transmission represent another important contribution to phylogeographic incompatibilities, with lineages following different routes between the source and the final location. In model (i), an additional source of incompatibility is provided by bidirectional spread, with lineages bouncing back and forth or wandering between distant locations.

The upper panel of Fig. 2 reveals that the incompatibility between simulated trees increases with both recombination rate and migration rate, at least for low/moderate rates of migration. This is to be expected, since increased recombination implies more different trees, while low migration leads to less spatial transitions along branches and therefore more similar geographic histories.

In most models, changes in recombination rates have a much stronger impact on phylogeographic incompatibilities than changes in migration rates. This can be explained by the fact that recombination is necessary to actually observe a difference in migration histories. Without any topological variation of the tree, the phylogeographic reconstruction would not differ and any change in migration patterns would be undetectable. In fact, incompatibilities are very small for recombination rates ρ<1 and increase steeply for larger values of *ρ*. Incompatibilities tend to be small as well for low migration rates μ≪1.

As outlined above, migration patterns have a major impact on incompatibilities, depending on the migration rate and the specific measure of incompatibility. Incompatibilities tend to be small if there is a single source population (Fig. 2-ii), since this imposes strong constraints on the migration histories and on the origin of the ancestors of the sample. Scenarios with longer transmission chains (Fig. 2-iii) or more complex patterns of transmission (Fig. 2-iv) lead to a slight increase in differences among phylogeographies and a more complex dependence on migration rates. Finally, in scenarios with multiple bidirectional migration patterns, migration histories can be very different, hence incompatibilities tend to be much larger (Fig. 2-i) and to increase monotonically but weakly with migration rates.

In some scenarios, different measures of incompatibilities show strikingly different trends. This is due to the fact that they capture different components of the signal. As an example, widespread differences in recent geographical history would give rise to a much stronger signal on MASPG than on MRCA incompatibilities.

### Analysis of the Initial Spread of the O/ME-SA/Ind-2001 FMDV Lineage Across the World

Foot-and-mouth disease is a viral disease affecting wild and domesticated cloven-hoofed animals (Alexandersen et al. 2003; Arzt et al. 2011a, 2011b) and it is one of the most economically relevant diseases of livestock worldwide (Knight-Jones and Rushton 2013). It is caused by the FMDV, a positive-sense single-stranded RNA virus belonging to the family *Picornaviridae*. This Aphthovirus has a relatively short (of about 8.2 kb) but highly variable linear genome, comprising the 5^′^UTR (untranslated region) followed by a single open reading frame (ORF) and the 3^′^UTR region. The ORF encodes for 12 proteins with different functions: the leader protease (L^pro^), four capsid proteins (VP1, VP2, VP3, and VP4) and nonstructural proteins (2A, 2B, 2C, 3A, 3B, 3C, and 3D) (Jackson et al. 2003). It provides an interesting case study for phylogeographic incompatibilities, since it has a nontrivial geographic structure, being spread out across host populations worldwide (Di Nardo et al. 2011; Brito et al. 2017), and a high recombination rate, especially between structural and nonstructural proteins (Carrillo et al. 2005; Jackson et al. 2007).

**Fig. 3.**
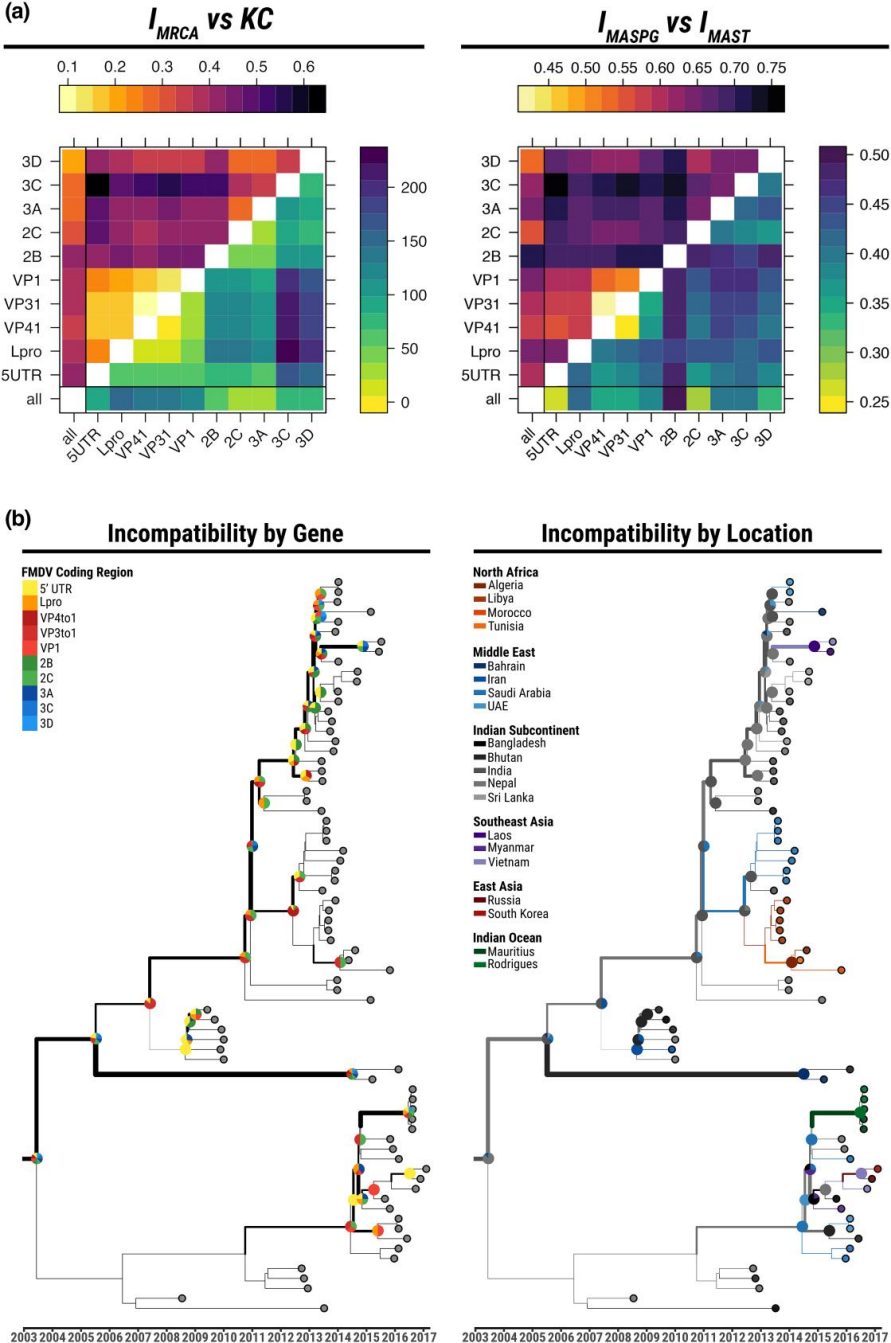
Phylogenetic and phylogeographic incompatibilities estimated for the FMDV O/ME-SA/Ind-2001 epidemics. Panel a—Comparisons of various incompatibility measures between phylogeographies reconstructed from segments of the FMDV O/ME-SA/Ind-2001 genome. In the plot on the left, $I_{MRCA}$ (upper triangle) is compared to *KC* (lower triangle). In the plot on the right, $I_{MASPG}$ (upper triangle) is compared to $I_{MAST}$ (lower triangle). Panel b—Illustration of the incompatibilities along the maximum clade credibiltiy (MCC) tree. The line-width of each branch represents the total amount of phylogeographic MRCA incompatibilities between all trees built from individual genomic regions and the whole-genome tree. Pie charts represent the distribution of MRCA incompatibilities per genomic region (left) and per country (right) on the corresponding nodes. Branch colors on the right topology represent the phylogeography reconstructed from the WGS alignment.

Whole-genome sequences (WGSs) (n=74) of a globally circulating FMDV lineage, the O/ME-SA/Ind-2001, were provided by the World Reference Laboratory for FMD (WRLFMD) at the Pirbright Institute, UK (Bachanek-Bankowska et al. 2018). Discrete phylogeographic reconstruction was performed on most loci along the genome (10 proteins), as well as on the whole genome sequence (WGS) (supplementary Fig. 2, Supplementary Material online). The phylogenetic and phylogeographic incompatibilities are illustrated in Fig. 3.

The two measures of phylogeographic incompatibility are only partially related. However, this is true for usual phylogenetic incompatibilities as well, as can be seen comparing the left and right panel of Fig. 3a.

Our results show that the portions of the genome coding for the capsid structure (comprising the structural proteins from VP1 to VP4) have highly coupled histories, both phylogenetically and geographically. This fits with the well-known fact that this region exhibits a relatively low recombination rate (Carrillo et al. 2005; Jackson et al. 2007). The nonstructural genes at the 3^′^ end of the genome (i.e. 2C, 3A, 3C, and 3D) also share similar phylogenetic and geographic histories. The majority of incompatibilities are located along the tree trunk of the main sublineage, as illustrated in Fig. 3b: they are mostly related to differences in reconstructed virus movements within the Indian subcontinent, as well as variability in the timing of transmission of different genomic regions to the Middle-East.

Some genomic regions share similar phylogenies, but clearly distinct phylogeographies. This is the case e.g. for the 5^′^ UTR versus 2B-3A and for 2B versus 2C-3A, according to the MRCA measures estimated. In fact, topological similarity might not always reflect compatible geographical histories, and this relation is not reflected in the phylogenetic distances: for example, the WGS reconstruction is phylogenetically similar to the one from the L-protease (∼16% of the ORF), but the geographical histories are quite different.

It is important to note that the phylogeography created using the WGS does not provide a good summary of the phylogeographies of the individual genomic regions, and in fact it only resembles the phylogeographies from the genome segments comprising the 2C and 3A proteins, which tend to be quite dissimilar to those of the rest of the sequences (supplementary Fig. 2, Supplementary Material online). This could be due to the effect produced on the WGS topology by recombinant viruses (i.e. the O/BAR/15/2015 and O/BHU/3/2016) (Bachanek-Bankowska et al. 2018), which provide the strongest contribution to incompatibilities (as it is evident from their long branches in Fig. 3b).

It is therefore important in FMDV evolutionary studies to take account of geographic as well as phylogenetic differences when comparing reconstructed evolutionary histories of distinct genomic regions outside of the capsid.

### Spatial Evolution of Genomic Segments of Influenza B

Influenza B virus is a segmented virus in the family *Orthomyxoviridae*. It is a major human pathogen, although it causes a lesser threat to public health than the related influenza A virus. Influenza B virus has a short positive-sense single-stranded RNA genome (of about 15 kb) organized in eight linear segments (Bouvier and Palese 2008). Reassortment of these segments plays a major role in the evolution of the virus, hence influenza B represents an interesting case study for phylogeographic incompatibilities in reassorting pathogens. Two main lineages (denoted as Victoria and Yamagata) are recognized based on differences in the hemagglutinin protein. It has been previously shown how these lineages evolved under different dynamics (Langat et al. 2017).

**Fig. 4.**
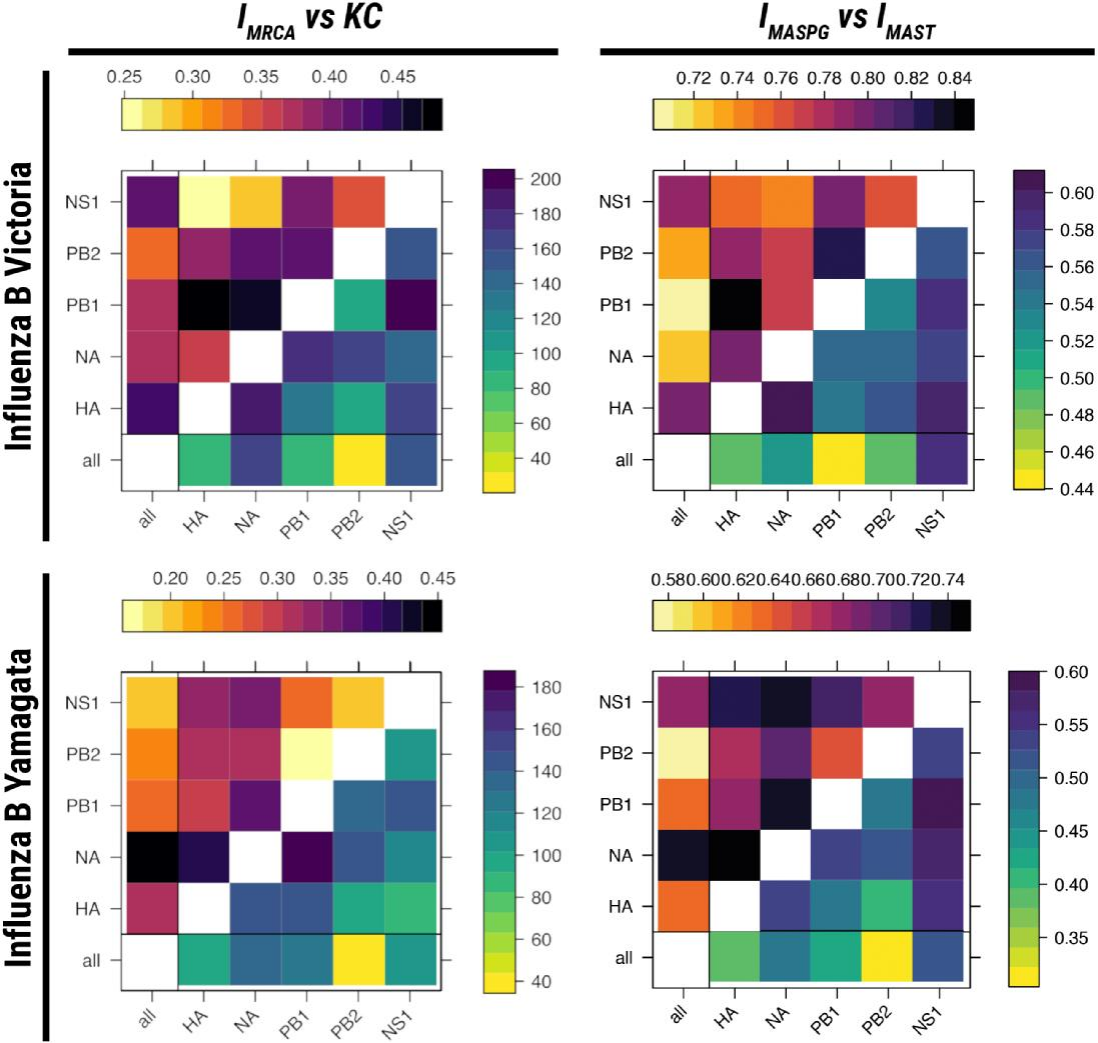
Comparisons of various incompatibility measures between phylogeographies reconstructed from segments of the influenza B genome for both the Victoria and Yamagata lineages. On the left, $I_{MRCA}$ (upper triangle) is compared to *KC* (lower triangle) for the Victoria (upper plot) and Yamagata (lower plot) strains. On the right, $I_{MASPG}$ (upper triangle) is compared to $I_{MAST}$ (lower triangle) for the Victoria (upper plot) and Yamagata (lower plot) strains.

We analysed a set of 242 sequences (122 of the Victoria lineage, and 120 of the Yamagata) from a recent study of worldwide evolution of these Influenza lineages (Bedford et al. 2015). These sequences include five genes (HA, NA, PB1 and PB2, and NS1 protein) located on different segments and therefore reassorting freely among them. Phylogenetic and phylogeographic incompatibilities among these segments are illustrated in Fig. 4 for both lineages. We also reconstructed and compared the phylogeographic history of the joint sequences of all these genes (∼8.6kb) (supplementary Figs. 3 and 4, Supplementary Material online).

**Fig. 5.**
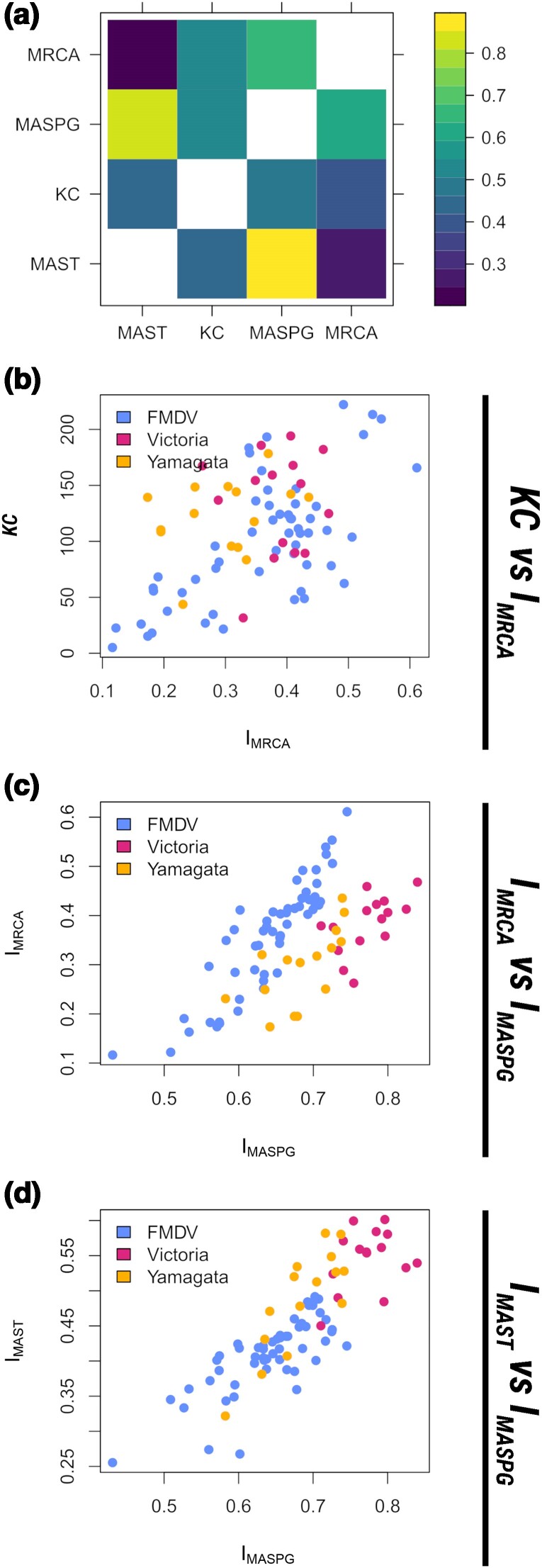
Illustrations of the correlations between the measures investigated by this study. a) Correlations between incompatibility measures. The upper triangle shows the Pearson correlation, and the lower triangle shows the Spearman correlation. b, c, d) Scatter plots illustrating the correlations between incompatibility measures for the three viruses whose phylogeographies were investigated in this paper.

Despite free reassortment between all segments, the structure of incompatibilities follows some clear patterns. Phylogeographic incompatibilities between segments are stronger for the Victoria strain, while for Yamagata these are comparable to the ones of a recombining virus such as FMDV (in Fig. 5b–d the Victoria strain can be seen to exceed Yamagata in most inter-segment distance measures). This is partly a result of the large impact of reassortment on the phylogenetic trees of the Victoria lineage.

There is a strong mismatch between the evolutionary histories of the two proteins that are the most important in the immune response against the virus (i.e. of HA and NA). The phylogenetic histories of HA and NA are highly divergent in the Victoria lineage, but the difference between their geographical histories is less extreme. On the other hand, the geographical spread of HA and NA in the Yamagata lineage followed very different routes, despite intermediate levels of reassortment. The geographical history of NA in this lineage is, in fact, markedly different from all other genes.

**Fig. 6.**
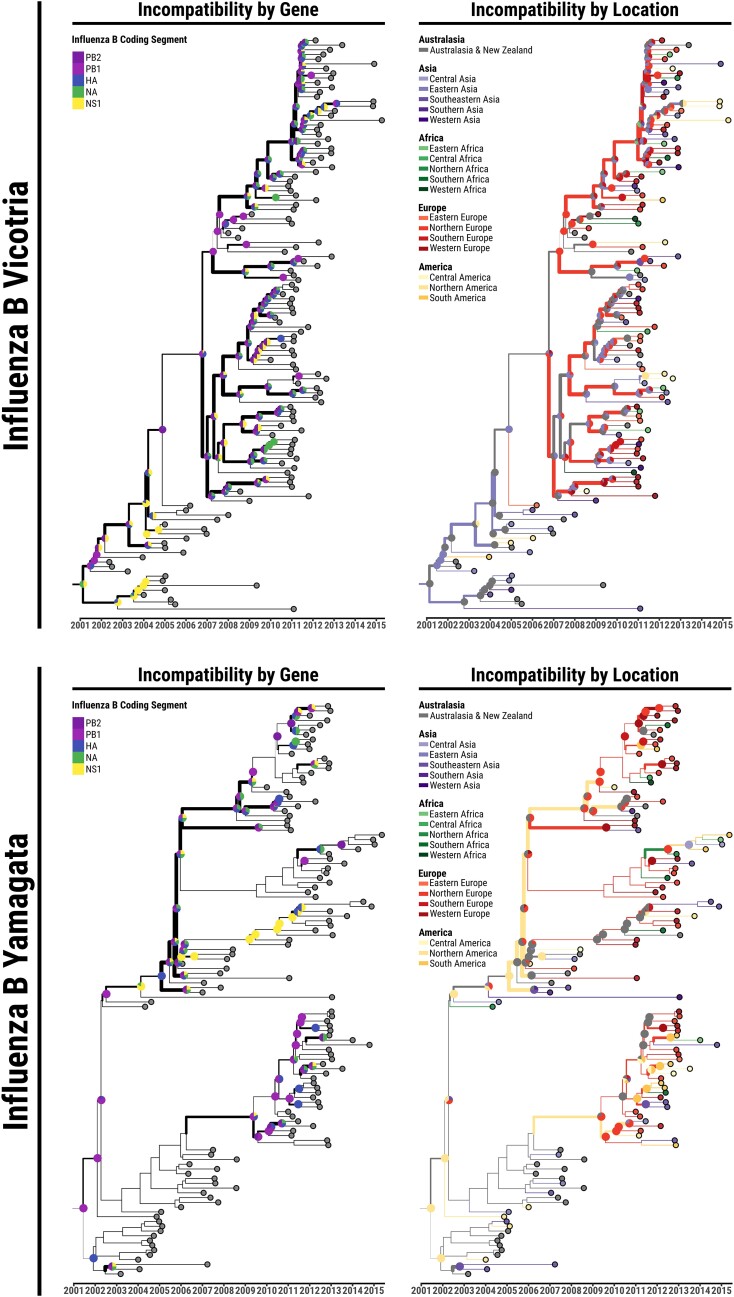
Illustration of the incompatibilities along the MCC tree for the Influenza B Victoria and Yamagata lineages epidemics. The line-width of each branch represents the total amount of phylogeographic MRCA incompatibilities between all trees built from individual genomic regions and the whole-genome tree. Pie charts represent the distribution of MRCA incompatibilities per genomic region (left) and per UN geographic region (right) on the corresponding nodes. Branch colors on the right topology represent the phylogeography reconstructed from the joint sequences alignment of all the genes investigated (∼8.6 kb).

Clear patterns of incompatibility differ between the two lineages, as shown also in Fig. 6. PB1 and PB2 phylogeographies are closely related for Yamagata but very different for Victoria. HA and NA phylogeographies are related to NS1 for Victoria, but the three genes present very different phylogeographies for Yamagata. Hence, the Victoria and Yamagata lineages differ not only in the tree topology, but also in the way different genes are spread geographically.

For the Victoria lineage, different geographical histories in the basal lineages of the tree correspond to different movements among East Asian and Australasian regions, whilst in more recent years viruses are reconstructed to spread in different ways between Asia and Europe, with the trunk more concentrated in Asia for NA and HA and in Europe for the other segments. For the Yamagata lineage, whilst the clade 2 (i.e. B/Massachusetts/02/2012 clade) shows little evidence of incompatibility between geographical histories, the spatial evolution of different genomic segments along the trunk of clade 3 (i.e. B/Wisconsin/1/2010 clade) clearly follows different routes of epidemics across Europe, America, and East Asia/Australia.

In addition, for the Influenza B case, the phylogeographic reconstruction from all genes together is not a reliable description of the evolutionary histories of individual genes. For both lineages, the overall phylogeny and phylogeography resembles mostly the one of PB2 and it is strongly incompatible with the geographic history of the NS1, hemagglutinin (in the Victoria lineage) and of the neuraminidase (in the Yamagata one) (supplementary Figs. 3 and 4, Supplementary Material online).

### Genetic Variation and Spatial Movement of Uralic-speaking Populations

We demonstrate the applicability of our incompatibility measures to eukaryotic organisms and to linguistic data, using genetic and lexical data from a study of Uralic languages and the ethnic groups who speak them (Tambets et al. 2018). Uralic is a language family containing languages spoken in parts of northern Eurasia, including Hungarian, Finnish, and Estonian among others.

**Fig. 7.**
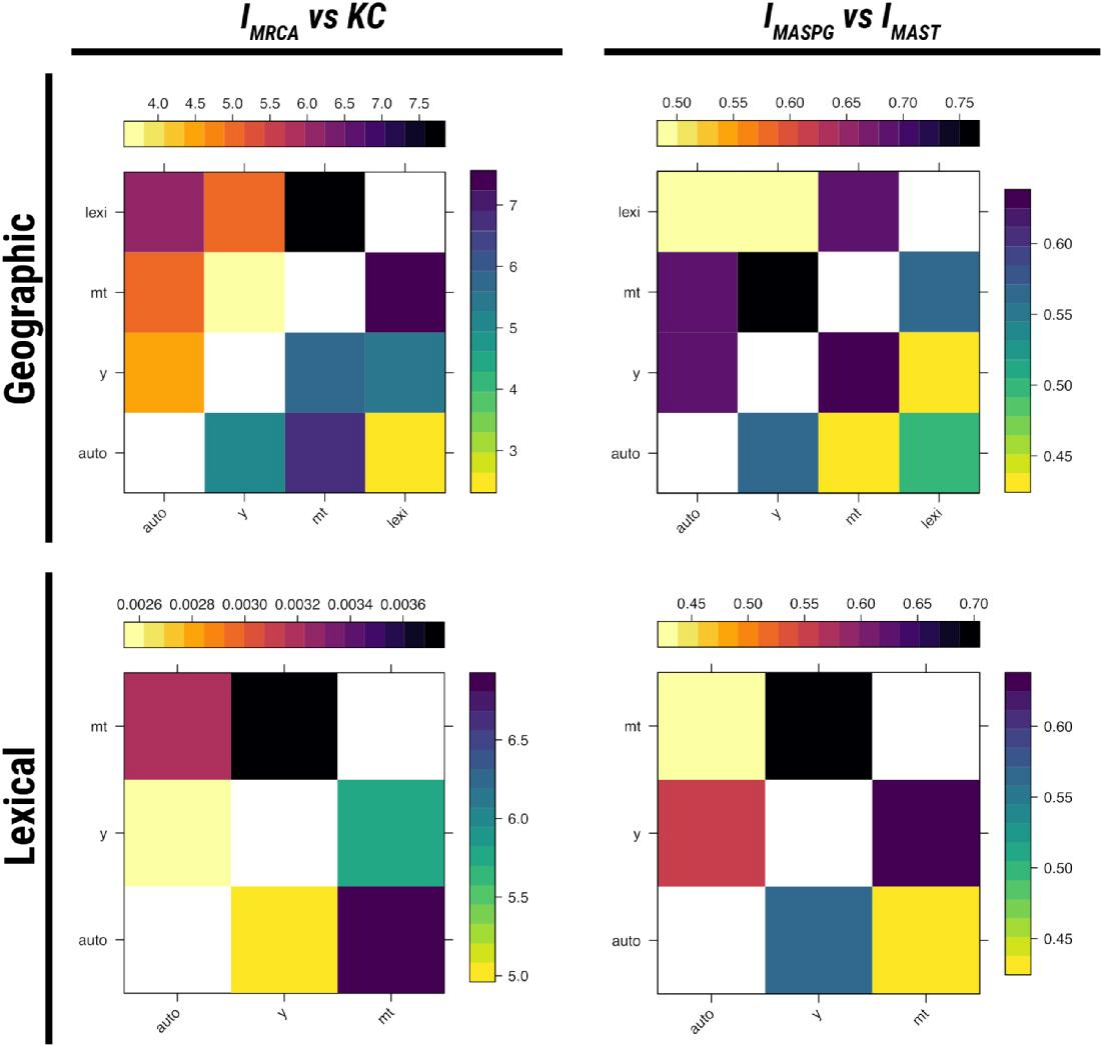
Top row: phylogenetic and phylogeographic incompatibilities between autosomal, Y-chromosome, mitochondrial, and lexical phylogenies. The trait that varies continuously along the phylogeny is the geographic location of the corresponding populations in Northern Eurasia. Bottom row: phylogenetic and phylolinguistic incompatibilties between autosomal, Y-chromosome, and mitochondrial phylogenies. The trait that varies continuously along the phylogeny is the location in two-dimensional projection of the lexical space of Uralic languages.

There are two ways to incorporate linguistic data into a phylogenetic analysis: either by adding linguistic characters to the trees (analogously to geographic characters), or using linguistic data to define sequences on which a tree can be inferred, thereby creating an ersatz phylogeny. We demonstrate the use of our incompatibility measures using both approaches in Fig. 7. In the top row, the phylogeographic tree inferred on lexical data is included among trees inferred on the genetic data from the Y-chromosome, mitochondrial, and autosomal DNA. We use only data from males, so all trees have comparable leaves. We display the phylogenetic and phylogeographic incompatibilities for these trees. In the bottom row, we replace the geographic space with a two-dimensional projection of lexical characters in the tree inference process, using Multi-Dimensional Scaling (MDS). Then we apply our incompatibility measures treating the lexical space as if it would be a geographic one, using a lexical distance measure on the MDS projection instead of the geographic distance. This yields a measure of phylolinguistic incompatibility.

For the phylogeographic incompatibilities, we find that the pattern in the values of $I_{MRCA}$ diverges from that of the KC distance more than $I_{MASPG}$ differs from $I_{MAST}$. In all cases, we see that the lexical tree is closer to the Y-chromosome tree than the mitochondrial one. This suggests that Uralic languages follow patterns of transmission that are more patrilinear than matrilinear in the Uralic-speaking populations (Gomes et al. 2017; Lansing et al. 2017). For the phylolinguistic incompatibilities, we again find that the pattern in the values of $I_{MRCA}$ diverges from that of the KC distance more than $I_{MASPG}$ differs from $I_{MAST}$. Further, in this case there is no relationship that can be found in all four measures, suggesting that these measures are highly divergent in the phylolinguistic case. This could be due to sensitivity to different parts of the tree—in fact we find that the trees differ at the root, so sensitivity to the root will have large effects on incompatibility measures (see supplementary Fig. 5, Supplementary Material online).

## Discussion

We have here presented two measures of phylogeographic concordance or incompatibility, $I_{MRCA}$ and $I_{MASPG}$. The first is derived from comparison of the geographical status of all the MRCAs of pairs of leaves. The second is derived from maximum agreement sub-phylogeographies. Both of these measures can be used to define metrics on the space of phylogeographies.

These incompatibility measures can be applied to any phylogeography, and more generally to any phylogeny where each tip has been assigned a geographical discrete location or any other continuous or discrete trait that is independent of the sequence. Such traits could include the host species for pathogens, environmental variables for species living in a varied range of environments or, as demonstrated, linguistic features of human populations. The natural assignment method is parsimony as pioneered by Maddison (1991, 1995), or Bayesian approaches (Lemey et al. 2009, 2014) such as those implemented in BEAST (Suchard et al. 2018), but our measures are independent of the reconstruction method used and on the specific trait. Our approach can also compare multiple trees inferred from different origins, e.g. phylogenetic vs linguistic trees, provided that the tips have the same geographical labels (representing e.g. different populations).

Phylogenetic congruence does not imply absence of recombination. In fact, there are many recombination events that do not change the tree or its topology (Ferretti et al. 2013). For similar reasons, phylogeographic incompatibilities cannot always be detected if only ancestral node states are known or reconstructed, since they ignore back-and-forth migration within a specific branch.

The two measures we define in this study are correlated but by no means identical. In fact, they are sensitive to different aspects of phylogeographic incompatibilities. We present these two particular measures in detail due to their naturalness and ease of computational implementation. There are however alternative possibilities, including extensions of tree distances other than the KC and MAST distances. One interesting generalization would be to include geographical information in SPR distances (Allen and Steel 2001; De Oliveira Martins et al. 2014). However, such a generalization requires many arbitrary decisions in how internal node states are treated, and its values are challenging to compute. In practice, $I_{MRCA}$ is simple but effective and could be the primary choice for phylogeographic incompatibilities.

The computational time for both these incompatibility measures scales quadratically with the number of leaves, hence they can be applied to large phylogeographies but at a computational cost. A simplified approach is to estimate the $I_{MRCA}$ incompatibility on a random subset of pairs of leaves. This simplified measure is unbiased compared to the true $I_{MRCA}$; this approach could even provide a measure of uncertainty via bootstrap. On the other hand, it is not valid to estimate the $I_{MASPG}$ incompatibility on a random subphylogeography since this approach is downward biased, similarly to what happens for the MAST of random subtrees.

Further work could explore extensions to these methods such as allowing incomplete geographical metadata for the samples, constraints on the geographical assignments of internal nodes, or weighting geographical incompatibilities by their location on the phylogeny. We limited our simulations to neutral variation between pairs of loci; further computational work could explore the possibility of detecting local adaptation on specific genomic regions through the genome-wide pattern of phylogeographic incompatibilities, since adaptation creates an association between localities and alleles, and it is likely to distort the geographical pattern around the selected loci.

One important limitation of our approach is that we consider distinct phylogeographies, instead of a phylogeographic network or a geographically labeled Ancestral Recombination Graph (ARG). This simplification is rooted in current approaches to phylogeographic inference. In fact, both phylogenetic trees and phylogeographies are usually inferred from sequence data and geographical metadata; most often, inference is performed separately for each locus, resulting in posterior distributions with unknown correlations between loci. To account for uncertainties in the inference, we defined a measure of incompatibility over both posterior distributions of phylogeographies which is conservative with respect to potential correlations.

Eventually, a full stochastic model of sequence evolution, birth–death, transmission, recombination, and geography should be developed for joint inference at multiple loci, following some recent developments (Guo et al. 2022; Stolz et al. 2022). This conceptual and computational challenge would allow full analysis of divergent geographical histories in genomes. The model of Dialdestoro et al. (2016) has the above components except transmission and geography. Incorporating transmission and geography is a technical challenge, with scaling to large data sets being a particularly difficult hurdle. Recent developments enable the inference of ARGs as collection of related trees for large genomes and large numbers of sequences (Kelleher et al. 2019), but it is unclear how the model can be extended to accommodate uncertainties and trait reconstruction. However, even a complete statistical model will not make the present work redundant, in the same way that phylogenetic incompatibilities are still useful even when it is possible to infer ARGs. In fact, any approach to statistical phylogeography is faced with the need to define some kind of incompatibility between phylogeographies. In its simplicity, the approach outlined here is to our knowledge the first method for systematic comparison that provides definition of distance between reconstructed phylogeographic histories.

## Materials and Methods

### Coalescent Simulations

To investigate the dependence of $I_{MRCA}$ on evolutionary parameters, we implemented a simulator of genealogies with migration and recombination. This simulator generates ARGs based on structured coalescent simulations in MASTER 6.1.1 (Vaughan and Drummond 2013), implemented in BEAST2 2.6.1 (Bouckaert et al. 2019). The simulation uses an island model of population structure with three populations of 20 individuals each and different patterns of migration between all populations (as illustrated in Fig. 2). In practice, the structured coalescent model is implemented backward in time as a combination of coalescence events (two lineages within the same island that coalesce into one) with rate 1, migration events (a lineage that changes island according to the reverse of an arrow in Fig. 2) with rate *μ* for each arrow, and recombination events (a lineage that splits into two lineages within the same island) with rate *ρ*. The simulations output geographically labeled ARGs for pairs of loci. We converted the ARGs into pairs of phylogeographies, one for each locus, and calculated their pairwise $I_{MRCA}$ for each set of parameters. The mean $I_{MRCA}$ for each parameter combination is shown in Fig. 2.

### Phylogeographic Reconstruction

#### Spatial Migration of Influenza B Victoria and Yamagata Lineages and FMDV

In order to test our method to real evolutionary scenarios of pathogens, we compiled two different data sets of genome sequences from influenza B and FMD viral infections, which affect either human or livestock species. Reassortment events and recombination patterns have been described for both the viral infections along with their dynamics of geographical transitions (Carrillo et al. 2005; Jackson et al. 2007; Dudas et al. 2014; Bedford et al. 2015). For influenza B virus, we selected 122 and 120 unique genome sequences of, respectively, the Victoria (Vic) and Yamagata (Yam) lineages (Bedford et al. 2015; Langat et al. 2017), characterizing five out of the eight gene segments and encoding: the polymerase basic subunits 1 and 2 (PB1 and PB2), the hemagglutinin (HA) and neuraminidase (NA) glycoproteins, and the nonstructural protein 1 (NS1) (Bouvier and Palese 2008). For FMD we selected 74 WGSs characterizing the O/ME-SA/Ind-2001 lineage (Bachanek-Bankowska et al. 2018), extracting separate alignments for each of the four structural (VP1 to VP4) and six nonstructural (2A to 2C, and 3A to 3D) proteins, along with the leader polypeptide (Lpro) and both the 3^′^ and 5^′^ UTR alignments (Jackson et al. 2003).

Distinct time-resolved phylogeographic trees were inferred for each of the influenza B genes and FMDV proteins using BEAST 1.10.4 (Suchard et al. 2018). Virus evolution was modeled by defining: the substitution process by the HKY (Hasegawa et al. 1985) and GTR+*Γ*4 (Tavaré 1986) models for influenza B and FMDV, respectively; a log-normal relaxed molecular clock across branches (Drummond et al. 2006); a flexible Bayesian Skyride coalescent prior on trees (Minin et al. 2008). Phylogeographic patterns of lineage movement across geographic locations were reconstructed using a discrete-state continuous-time Markov chain (CTMC) process, and assuming a nonreversible transition model with a Bayesian stochastic search variable selection (BSSVS) procedure (Lemey et al. 2009). We define discrete traits as countries for FMDV and as geographic regions for influenza B, following the United Nations M49 Standard (https://unstats.un.org/unsd/methodology/m49/). The prior for the molecular clock rate was defined by a noninformative CTMC conditional reference prior (Ferreira and Suchard 2008) and a truncated Poisson prior was set for the number of nonzero rates, with all the remaining priors left at their default values. Markov chain Monte Carlo (MCMC) chains were run for 200 million iterations, sampling trees every 20,000 states. Mixing and convergence of the MCMC chain was assessed using Tracer 1.7.3 (Rambaut et al. 2018), ensuring sufficient sampling was achieved, where the Effective Sample Size (ESS) was at least 200 and not lower than 500 for each of the posterior parameters. Inference were based on the resulting 9,000 trees obtained after discarding the initial 10% of the chain as burn-in. Phylogeographic incompatibilities were assessed in the phylogeographic space of a subset of 100 trees uniformly sampled from the posterior set of reconstructed phylogenies, using an approximation of the Wasserstein metric as described above. The MCC summary trees were obtained with TreeAnnotator (distributed with BEAST 1.10.4).

#### Uralic Linguistic Ancestry

We retrieved previously published genotype data of autosomal chromosomes, mtDNAs and chrY of Uralic-speaking individuals (Tambets et al. 2018). Conventional $F_{ST}$ distance matrices derived for each genotype data along with linguistic (lexical) data were used to infer distinct phylogenies in FastME 2.1.6.1 (Lefort et al. 2015), which were subsequently re-projected in time using the *chronos* function implemented in the R package *ape* (Paradis and Schliep 2018). The linguistic data we used is composed of a set of 226 words translated into each language. Differences between languages are quantified depending on how many of these words are cognate (i.e. share a common origin) (Tambets et al. 2018). The linguistic space was build using the default MDS projection routines in R based on lexical distances. Continuous phylogeographies were reconstructed in BEAST 1.10.4 using the 2-dimensional lexical or geographic spaces as continuous traits, by fixing the previously reconstructed topologies and inferring spatial movement of traits only by using the relaxed random walk model that employs a Cauchy distribution to define branch-specific variation in dispersal rates (Lemey et al. 2010). All priors were left at their default values. MCMC chains were run for 200 million iterations, sampling trees every 20,000 states. Inference were based on the resulting 9,000 trees obtained after discarding the initial 10% of the chain as burn-in.

## Supplementary Material

msae126_Supplementary_Data

## Data Availability

The $I_{MRCA}$ and $I_{MASPG}$ functions for estimating differences between phylogeographies have been implemented in the *geotreespace* R package, which can be installed from its github repository (https://github.com/BenSinger01/geotreespace). All .xml files generated using BEAST and used for analysis have been deposited to Dryad and available from the following link https://doi.org/10.5061/dryad.15dv41p4s.
