# Supplementary figures and images for: Comparing Phylogeographies to Reveal Incompatible Geographical Histories within Genomes
Source: Mol Biol Evol. 2024 Jun 26;41(7):msae126. doi: 10.1093/molbev/msae126 (PMC11251493; doi:10.1093/molbev/msae126)

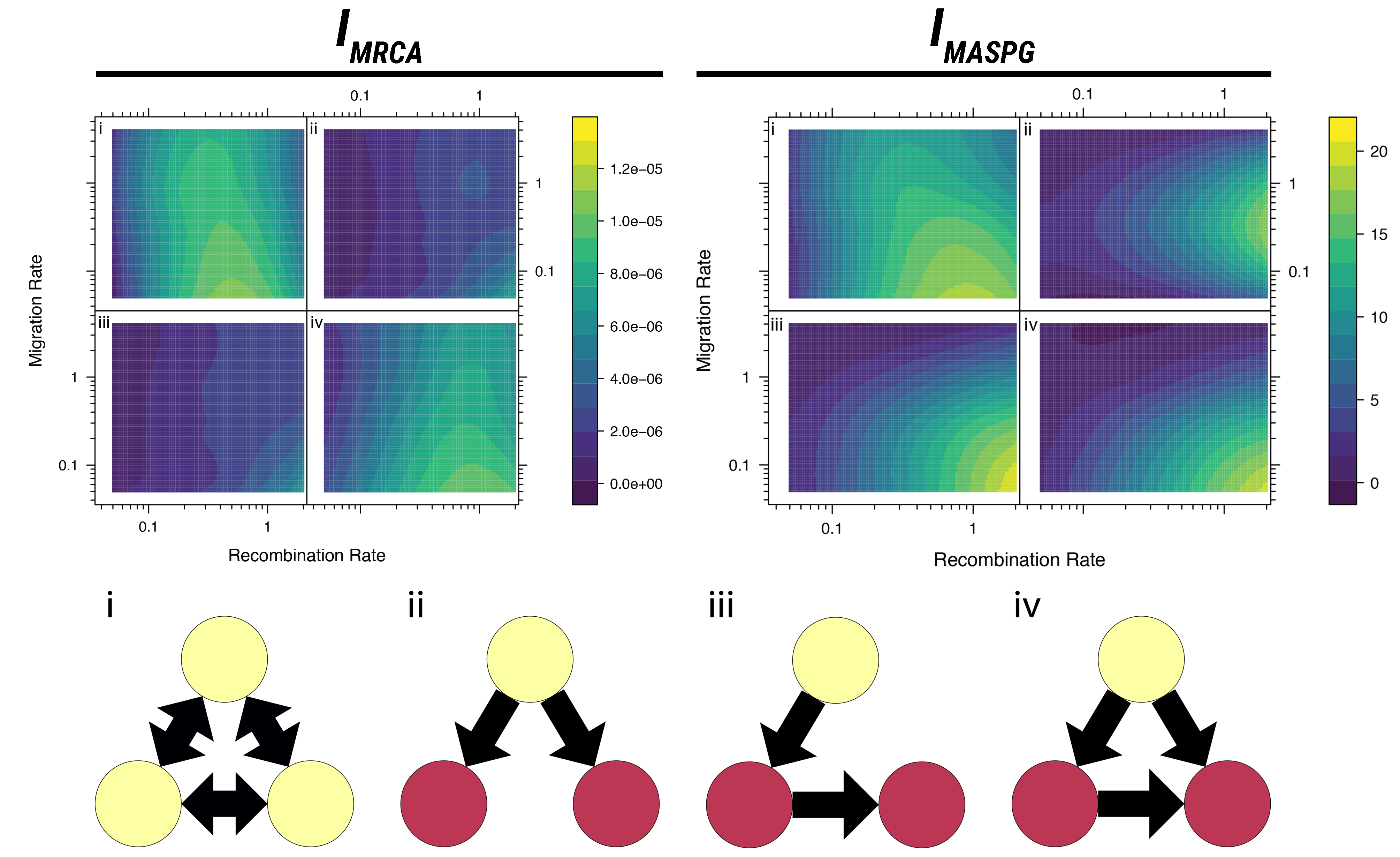

Supplement: msae126_Supplementary_Data [file msae126_supplementary_data.zip › FigSI6.tif]

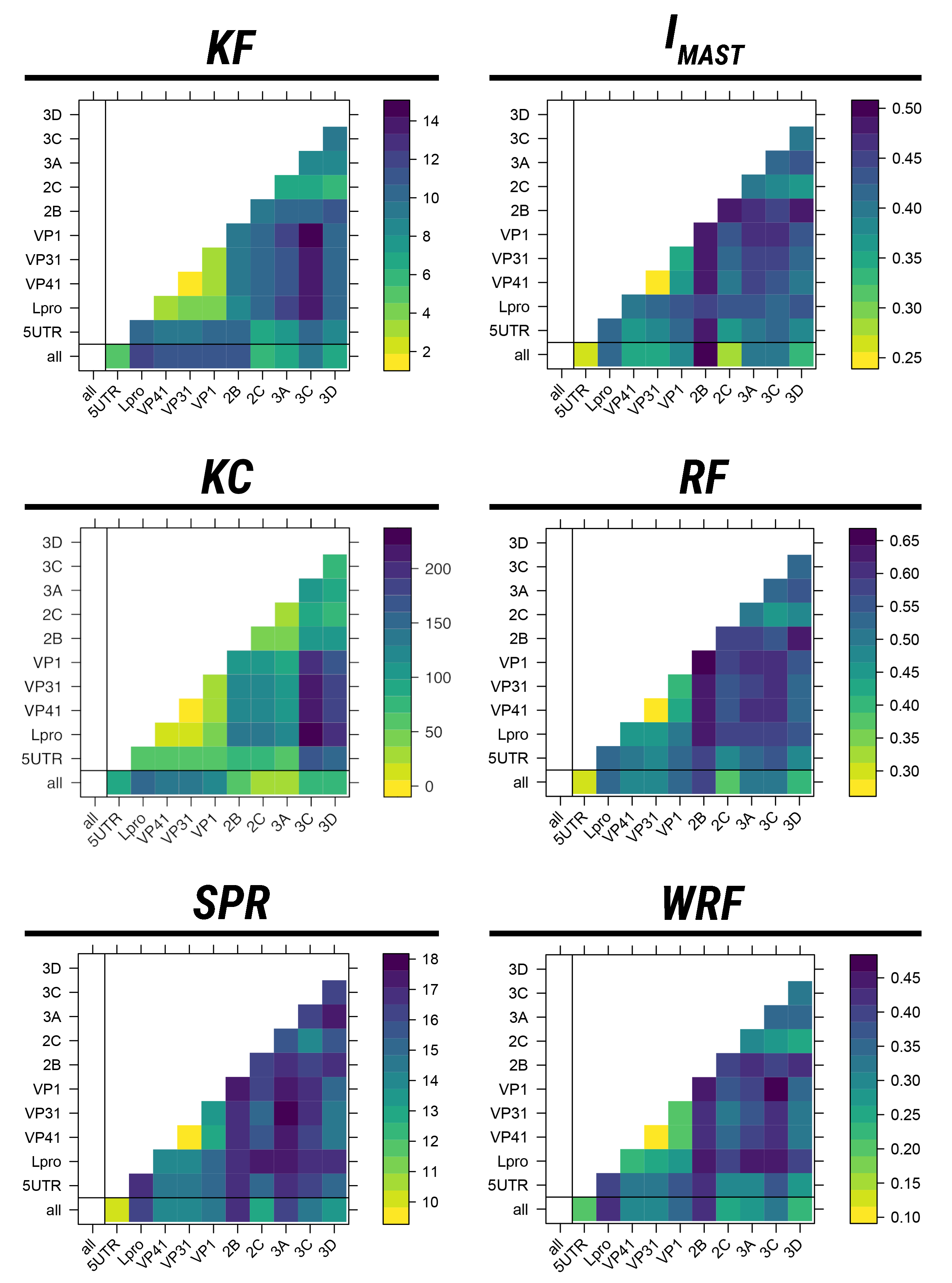

Supplement: msae126_Supplementary_Data [file msae126_supplementary_data.zip › FigSI7.tif]

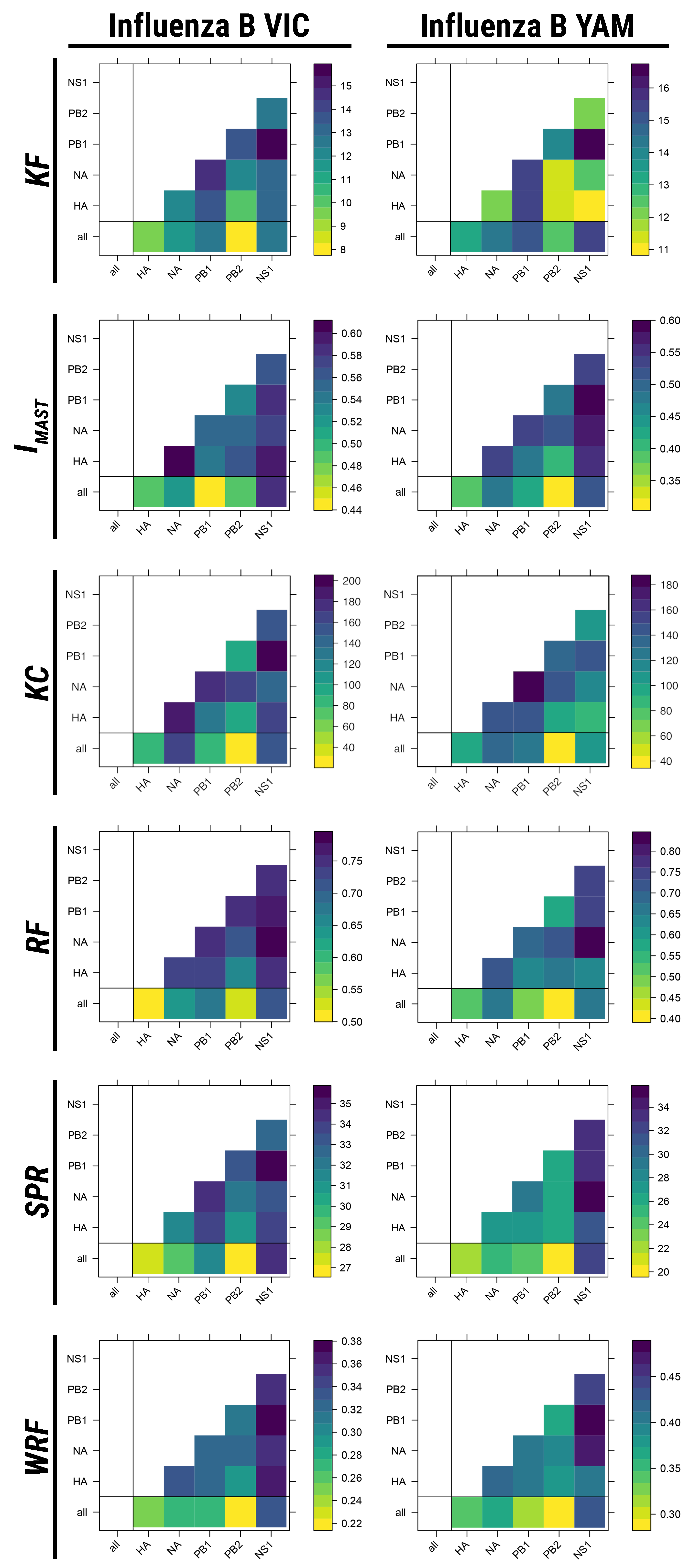

Supplement: msae126_Supplementary_Data [file msae126_supplementary_data.zip › FigSI8.tif]

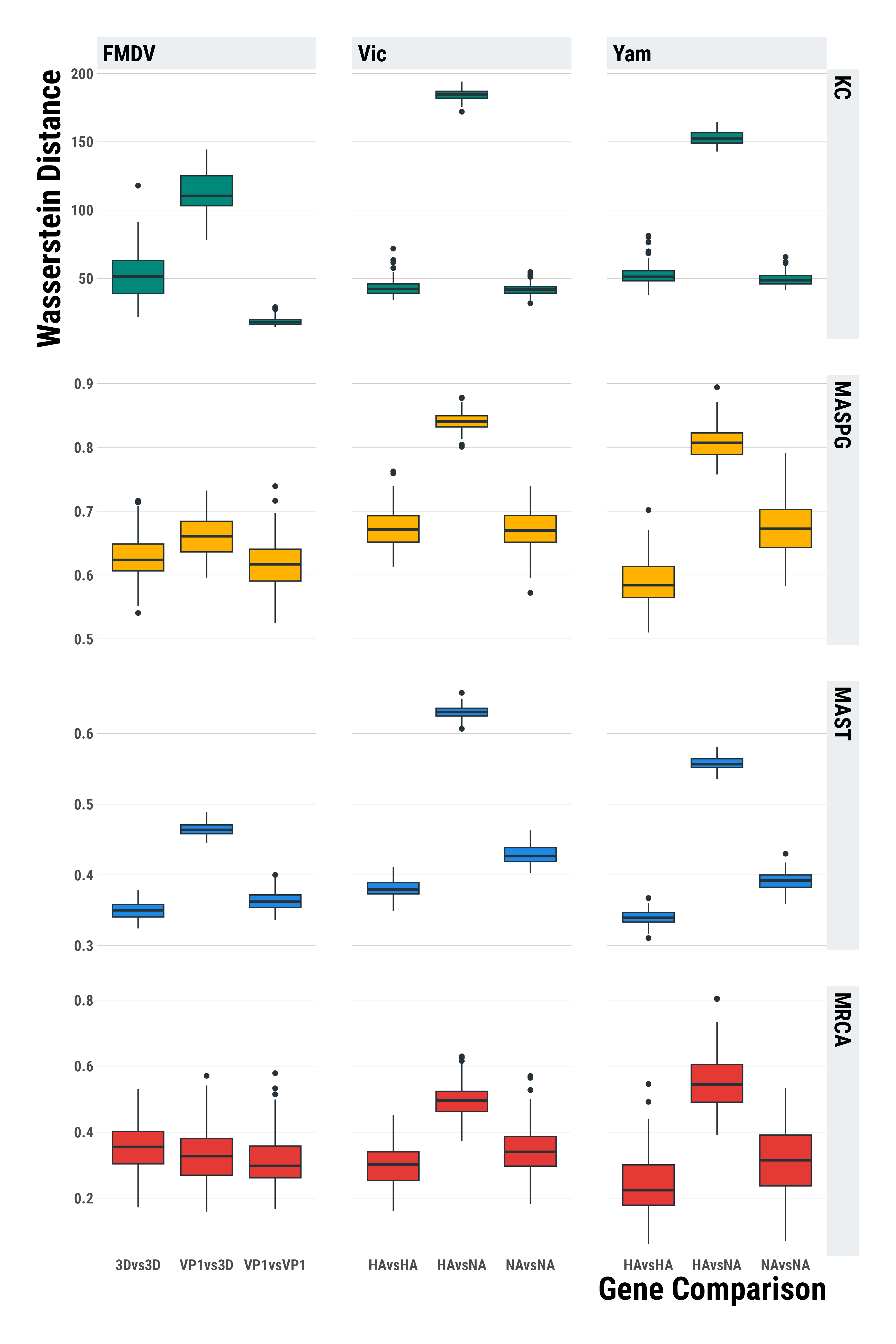

Supplement: msae126_Supplementary_Data [file msae126_supplementary_data.zip › FigSI9.tif]
